# Subtype Specificity of β-Toxin Tf1a from *Tityus fasciolatus* in Voltage Gated Sodium Channels

**DOI:** 10.3390/toxins10090339

**Published:** 2018-08-22

**Authors:** Daniel Oliveira da Mata, Diogo Vieira Tibery, Leandro Ambrósio Campos, Thalita Soares Camargos, Steve Peigneur, Jan Tytgat, Elisabeth Ferroni Schwartz

**Affiliations:** 1 Laboratório de Neurofarmacologia, Departamento de Ciências Biológicas, Universidade de Brasília, Brasília 70910-900, Distrito Federal, Brazil; daniel.oliveira.mata@gmail.com (D.O.d.M.); dtibery@gmail.com (D.V.T.); leandro.ambrosio@gmail.com (L.A.C.); 2 Departamento de Ciências da Saúde, Centro Universitário UDF, Brasília 70390-045, Distrito Federal, Brazil; thalitasoares@gmail.com; 3 Toxicology and Pharmacology, Department of Pharmaceutical and Pharmacological Sciences, University of Leuven (KU Leuven), P.O. Box 922, Herestraat 49, 3000 Leuven, Belgium; steve.peigneur@kuleuven.be (S.P.); jan.tytgat@kuleuven.be (J.T.)

**Keywords:** ion channel, Na^+^-channel modulator, neurotoxin, scorpion, *Tityus fasciolatus*, venom, voltage-gated sodium channels, β-scorpion toxin

## Abstract

Scorpion venoms are a complex mixture of components. Among them the most important are peptides, which presents the capacity to interact and modulate several ion channel subtypes, including voltage-gated sodium channels (Na_V_). Screening the activity of scorpion toxins on different subtypes of Na_V_ reveals the scope of modulatory activity and, in most cases, low channel selectivity. Until now there are approximately 60 scorpion toxins experimentally assayed on Na_V_ channels. However, the molecular bases of interaction between scorpion toxins and Na_V_ channels are not fully elucidated. The activity description of new scorpion toxins is crucial to enhance the predictive strength of the structural–function correlations of these Na_V_ modulatory molecules. In the present work a new scorpion toxin (Tf1a) was purified from *Tityus fasciolatus* venom by RP-HPLC, and characterized using electrophysiological experiments on different types of voltage-gated sodium channels. Tf1a was able to modify the normal function of Na_V_ tested, showing to be a typical β-NaScTx. Tf1a also demonstrated an unusual capability to alter the kinetics of Na_V_1.5.

## 1. Introduction

Scorpions belongs to the Arthropoda phylum, representing approximately 1.5% of the species present in Arachnidae class [1]. From the 160 species found in Brazil, the *Tityus* genus is considered the most important due to their medical relevance [1,2]. *Tityus fasciolatus* is found in the central region of Brazil, more precisely in the Cerrado biome causing accidents at those area [3,4,5].

Scorpion venoms are comprised of several compounds such as enzymes, free amino acids, heterocyclic components, peptides, and proteins [6]. The most studied components are peptides due to their abundance and distinct activity on ion channels [7]. These compounds acts on Na^+^, K^+^, Cl^−^, and Ca^2+^ channels changing their normal functioning [8]. Peptides acting on voltage gated sodium channels, also known as neurotoxins, are composed by 55–75 amino acid residues with four disulfide bonds and molecular masses between 6.5 and 8.5 kDa [6,9,10]. 

Scorpion toxins that act on voltage gated sodium channels (NaScTxs) are classified in α and β toxins, based on their effects and binding sites [10]. α–NaScTxs interact with site 3, altering their fast inactivation kinetics [7,10,11]. These toxins can be subdivided into classical α-toxins, anti-insect, and α-like toxins [6,11]. β–NaScTxs interact with site 4, causing an amplitude reduction in current and a voltage dependence activation shift to more hyperpolarized potentials [6,10]. These toxins can be subdivided into four different classes: antimammalian, antimammalian and anti-insect or β-like, anti-insect excitatory, and anti-insect depressant [6,11].

Voltage gated sodium (Na_V_) channels are essential for initiation and propagation of action potentials and conduction of electrical signals. Thereby, understanding the structure and functionality of these channels is very important in many aspects. The use of neurotoxins that act on Na_V_ channels was especially important for the elucidation of channel structure [12,13]. Na_V_ channels are transmembrane proteins formed by the principal α subunit of 260 kDa and the auxiliaries of β subunits [14]. The α subunit is formed by four domains (I to IV); each domain is composed of six transmembrane segments (S1 to S6) being the S4 known as voltage sensor [12,14]. The movement of S4 is responsible for channel opening, which enables the movement of ions [14,15].

Human Na_V_ channels are divided in nine subtypes (Na_V_1.1–1.9), each one with diverse role and location. Na_V_1.1, 1.2, 1.3, and 1.6 subtypes are found in the central nervous system (CNS), Na_V_1.4 in skeletal muscle, Na_V_1.5 in cardiac muscle/myocytes, and Na_V_1.7–1.9 in the peripheral nervous system (PNS) [16,17]. Channel-coding gene mutations are associated to many diseases described over the years and are called channelopathies [17,18].

The first peptide described from *Tityus fasciolatus* venom was Tf4, which was demonstrated to be an typical α-toxin [3]. The crude venom toxicity was also evaluated in cardiorespiratory system by electrocardiogram (ECG) in rats, showing that the dose of 2.3 mg/kg induces several cardiorespiratory alterations [4]. Guimarães et al. 2011, demonstrated that at 24 µg, *Tityus fasciolatus* venom causes piloerection, pain behavior, nasal and oral sharp, dyspnea, facial rash, excessive reflexes, and alteration of the blood profile in mice [19]. Posteriorly, the immunological response evoked by the *T. fasciolatus* crude venom was evaluated, and three toxins had their sequences described (Tf1, Tf3, and Tf4a) [5]. Posteriorly, a toxin named Tf2 was electrophysiological characterized using two-electrode voltage-clamp technique on human sodium channels subtypes, showing high selectivity to Na_V_ 1.3 [20]. This work aims to describe Tf1a and its activity on Na_V_1.1–1.7, Bg Na_V_, and Vd Na_V_ by electrophysiological experiments. 

## 2. Results

### 2.1. Toxin Purification 

**Figure 1:**
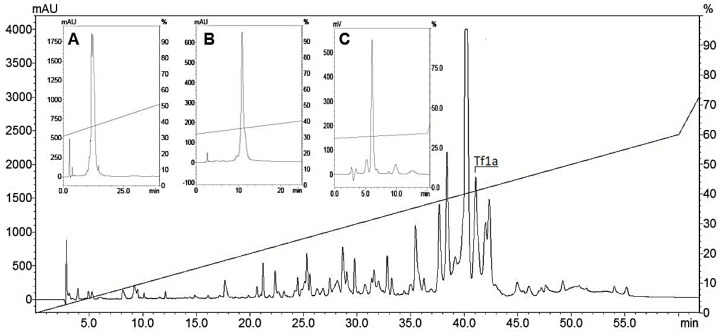
Chromatographic profile of *Tityus fasciolatus* venom. Chromatographic profile of 1 mg of *Tityus fasciolatus* crude venom using the RP-HPLC C18 column at 1 mL/min flow rate monitored at 216 nm. The fraction of interest, highlighted in the large image as Tf1a, eluted at 41.1 min (~41.1% acetonitrile). (**A**) 0.5%[B]/min. (**B**) Second purification step with 0.5%[B]/min at 45 °C. (**C**) Last purification step with 0.3%[B]/min at 45 °C.

*Tityus fasciolatus* crude venom (1 mg) generated 60 fractions after the RP-HPLC process, as previously shown in [20]. The fraction of interest corresponding to Tf1a was collected at 41.1 min (~41.1% of acetonitrile) and three extra steps of chromatography were performed to obtain the isolated peptide (Figure 1A–C).

### 2.2. Molecular Mass Evaluation and Sequence Determination

**Figure 2:**
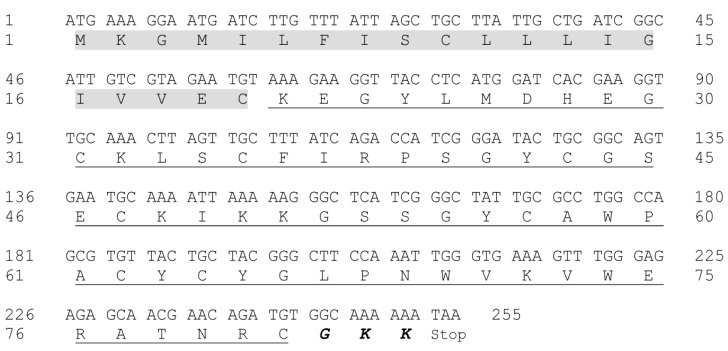
Nucleotide sequence of Tf1a precursor and the translated peptide. The nucleotide sequence was obtained by HiSeq (Ilumina, San Diego, CA, USA) and compared to the partial data obtained by ISD. Signal peptide is marked in gray, mature peptide is underlined, and the amidation signal is highlighted in bold and italic.

The average molecular mass of purified Tf1a was [M + H]^+^ = 6927.5 (Figure S1). Two partial sequences were obtained by the In Source Decay method (ISD); the complete sequence contains 32 amino acids residues (Figure S2). The results obtained were then compared to the RNA library extracted from *Tityus fasciolatus* venom gland (Figure S3) (unpublished data), showing that the fraction corresponds to a precursor sequence formed by 255 nucleotides. The translated peptide is composed of 81 amino acids residues, of which the first 20 amino acid residues are the signal peptide and the remaining 61 residues is the mature peptide (Figure 2). The sequence also shows the presence of GKK in the C-terminal, which is an amidation signal present in several scorpion sodium toxins (NaScTxs), and in some cases, such as in the toxin Ts1 from *Tityus serrulatus*, C-terminal amidation is important for NaScTx activity on sodium channels [9,21].

**Figure 3:**
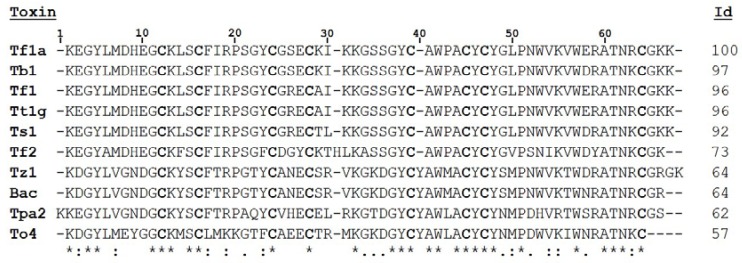
Multiple sequence alignment. Tf1a was aligned with other β-NaScTxs using CLUSTAL Omega. The cysteines (C) are shown in bold. The left column indicates the name and the right column shows the identity of each toxin with Tf1a. (*) identical residues; (:) conservative substitution; (.) semi-conservative substitution. Uniprot entry codes: Tb1: P56611; Tt1g: P0DMM8; Ts1: P15226; Tf2: C0HJM9; Tz1: Q2NME3; Bactridine 2 (Bac): P0CF37; Tpa2: P84631; To4: P60215. Amino acid numbering considered Tpa2 as reference.

The mature peptide sequence was compared and aligned with other sequences showing high identity with β-NaScTxs: 97% identity with Tb1 from *Tityus bahiensis*, 96% with Tt1g from *Tityus trivittatus*, 92% with Ts1 from *Tityus serrulatus*, 73% with Tf2, also from *Tityus fasciolatus*, 64% with Tz1 and Bactiridine 2 from *Tityus discrepans*, 62% and 57% with Tpa2 from *Tityus pachiurus*, and To4 from *Tityus obscurus* (Figure 3). Furthermore, the sequence has shown 96% identity with a toxin previously described by transcriptome analysis from *Tityus fasciolatus* called Tf1 [5]. Due to the similarity with Tf1 and according to [22] the toxin purified was named Tf1a

### 2.3. Electrophysiological Characterization 

**Figure 4:**
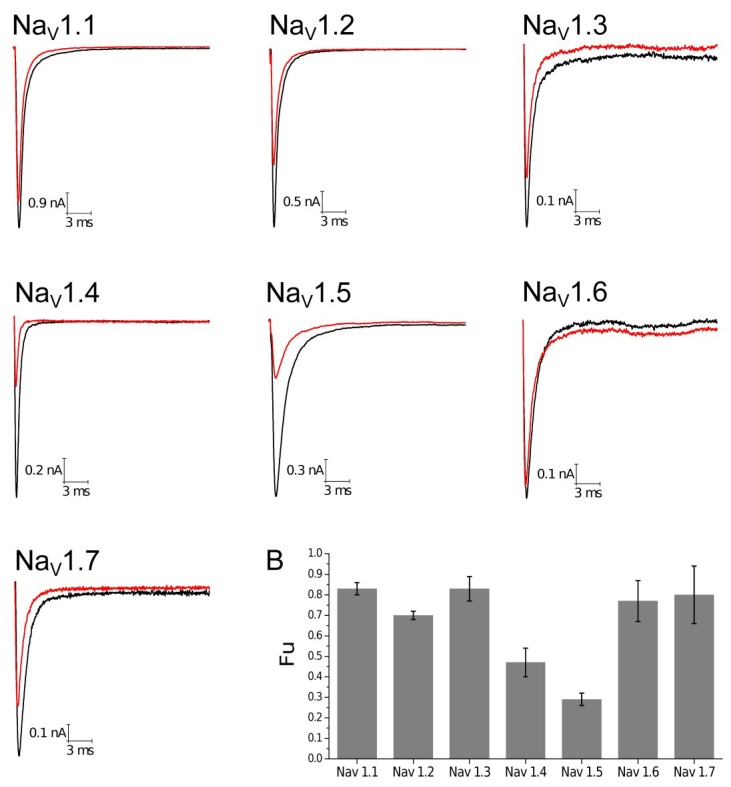
Current traces and Fraction uninhibited current (Fu) on human sodium isoforms. Sodium current traces were evoked in: Na_V_1.1 (−10 mV), Na_V_1.2 (0 mV), Na_V_1.3 (5 mV), Na_V_1.4 (10 mV), Na_V_1.5 (−25 mV), Na_V_1.6 (−5 mV), and Na_V_1.7 (0 mV). Red traces represent the presence of 100 nM Tf1a and black traces indicate control condition. (**B**) Graphical representation of the fraction uninhibited currents on human subtypes. The bars represent the standard error of mean.

All experiments were performed at 100 nM concentration based on the experiments done with other β-toxins, such as Ts1 and To4 [23,24], the number of observation equals four (*n* = 4) on each human Na_V_ subtype. The first parameter analyzed was the Fraction of uninhibited current (Fu). Na_V_1.4 and Na_V_1.5 were more altered by Tf1a, resulting in low values of Fu, 0.47 and 0.29, respectively, indicating a higher sensibility of these two subtypes to the toxin (Figure 4, Figure S4).

**Figure 5:**
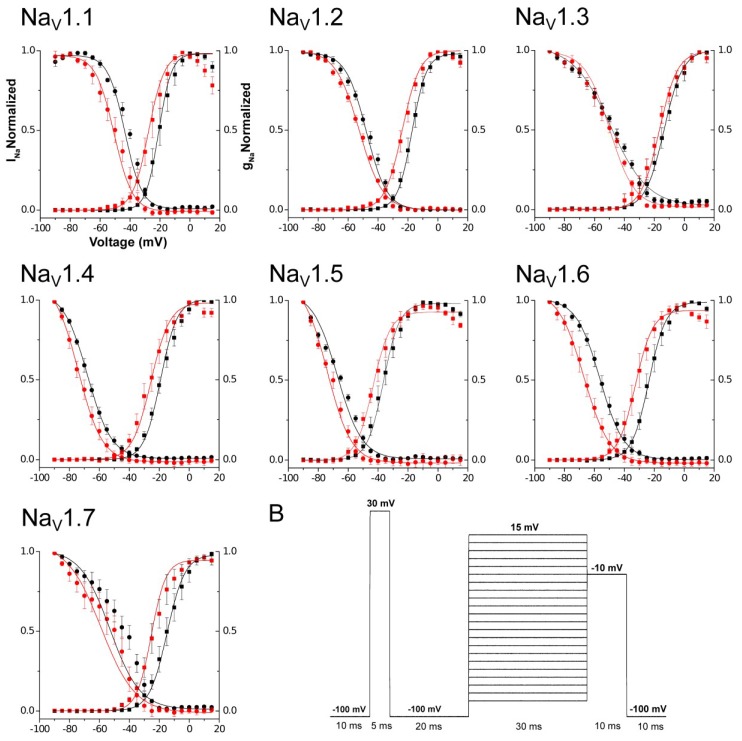
Open probability (ρO) and steady-state inactivation curves (SSI). The curves were generated by using the data of current and conductance normalized for each subtype tested using the Boltzmann function. Black squares represent control conditions for open probability (ρO) and red squares show conditions after the application of 100 nM of toxin at final concentration. Black circles represent control conditions for steady-state inactivation analysis (SSI) and red circles show conditions after the application of 100 nM of toxin at final concentration. The bars represent the standard error of mean. (**B**) Representation of the protocol used for the electrophysiological experiment. For Na_V_1.5, the interval time between prepulse and stimulation protocol was 50 ms. Sweep (start-to-start) interval of 2 s.

The second parameter was the evaluation of Open Probability (ρo) during the activation phase. The most affected subtype by the presence of Tf1a was Na_V_1.6 (ΔV_g_ = −9.00 ± 1.08 mV), followed by Na_V_1.7 (ΔV_g_ = −7.93 ± 1.16 mV), Na_V_1.4 (ΔV_g_ = −7.93 ± 3.32 mV), and Na_V_1.1 (ΔV_g_ = −7.08 ± 1.02 mV). Na_V_1.5 (ΔV_g_ = −6.28 ± 0.79 mV), Na_V_1.2 (ΔV_g_ = −6.09 ± 0.51 mV), and Na_V_1.3 (ΔV_g_ = −4.79 ± 1.04 mV) were less affected by Tf1a. Statistical analyses demonstrated significant activity on Na_V_1.1 (*p* = 0.0061), Na_V_1.2 (*p* = 0.0013), Na_V_1.3 (*p* = 0.0193), Na_V_1.5 (*p* = 0.0042), Na_V_1.6 (*p* = 0.0037), and Na_V_1.7 (*p* = 0.0065). Isoform Na_V_1.4 (*p* = 0.0971) did not demonstrate significant difference with a *p* > 0.05 probably due to the high standard error. All the results show the capacity of the toxin to induce a leftward shift, displacing a change in voltage dependence activation to more hyperpolarized potentials (Figure 5). These results were obtained in presence of a prepulse condition, due to the requirement for this stimulus in some β-scorpion toxins previously described [14]. Nevertheless, experiments were also conducted without prepulse and results were similar (Table S1).

Steady-State Inactivation was also investigated. The results found were very similar to those observed during the activating phase. The most affected subtype was Na_V_1.6 (ΔV_h_ = −11.41 ± 2.73 mV), followed by Na_V_1.1 (ΔV_h_ = −8.11 ± 1.94 mV), Na_V_1.7 (ΔV_h_ = −7.99 ± 2.54 mV), Na_V_1.4 (ΔV_h_ = −6.15 ± 1.06 mV), Na_V_1.5 (ΔV_h_ = −6.13 ± 1.83 mV), Na_V_1.2 (ΔV_h_ = −5.36 ± 0.61 mV), and Na_V_1.3 (ΔV_h_ = −2.93 ± 0.68 mV) (Figure 5). Statistical analyses demonstrated significance for activity on Na_V_1.1 (*p* = 0.00249), Na_V_1.2 (*p* = 0.0032), Na_V_1.3 (*p* = 0.0232), Na_V_1.4 (*p* = 0.0102), Na_V_1.5 (*p* = 0.0443), and Na_V_1.6 (*p* = 0.0250). Isoform Na_V_1.7 (*p* = 0.0516) did not demonstrate a significant difference with a *p* > 0.05, probably due to the high standard error. All data from ρO, SSI, and Fu are shown in Table 1 and Table 2.

**Figure 6:**
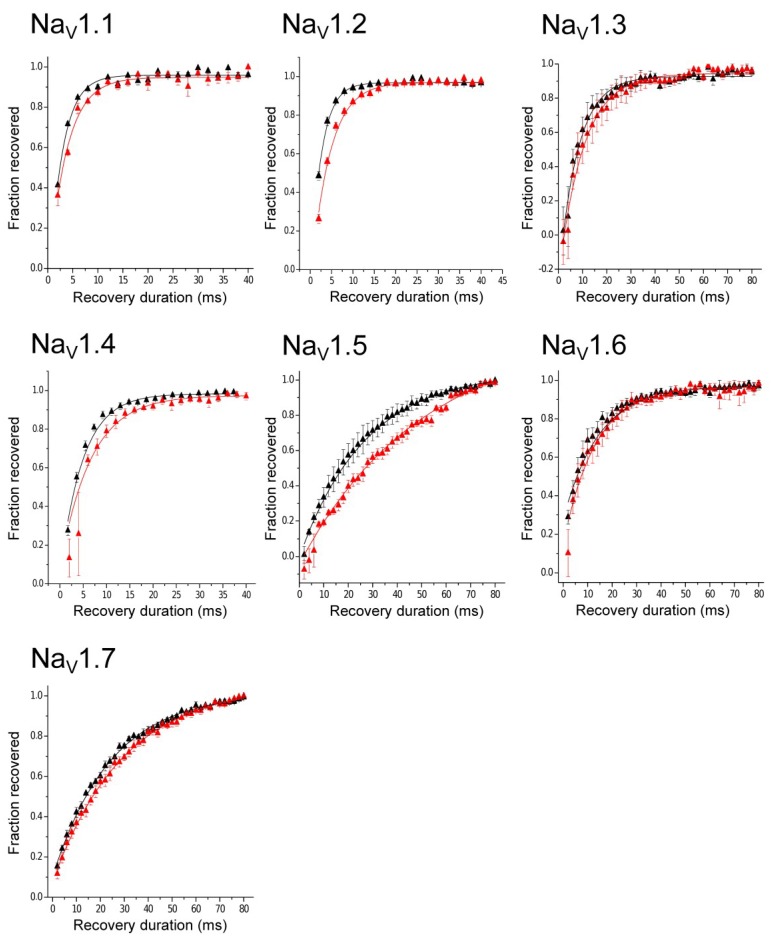
Recovery from inactivation curves. The curves were generated by the data obtained from recovery from inactivation, which were fitted using an exponential function. Open triangles represent control and red triangles show conditions after the application of 100 nM of Tf1a at final concentration. The bars represent the standard error of mean.

The next parameter evaluated was the Recovery from Inactivation. Statistical analysis showed that on Na_V_1.1, Na_V_1.2, and Na_V_1.5 there was a significant difference between control conditions and in the presence of toxin Tf1a, enhanced their time constant (τ) (Na_V_1.1 (*p* = 0.0257), Na_V_1.2 (*p* = 0.0238), and Na_V_1.5 (*p* = 0.0462)). On Na_V_1.3, Na_V_1.4, Na_V_1.6, and Na_V_1.7 there was no significant difference, with a *p* > 0.05. Tf1a showed the ability to affect the channel recovery, especially on Na_V_1.5 subtype, where the effect was prominent (Δτ = 20.11 ± 4.47 ms) (Table 3, Figure 6). 

**Figure 7:**
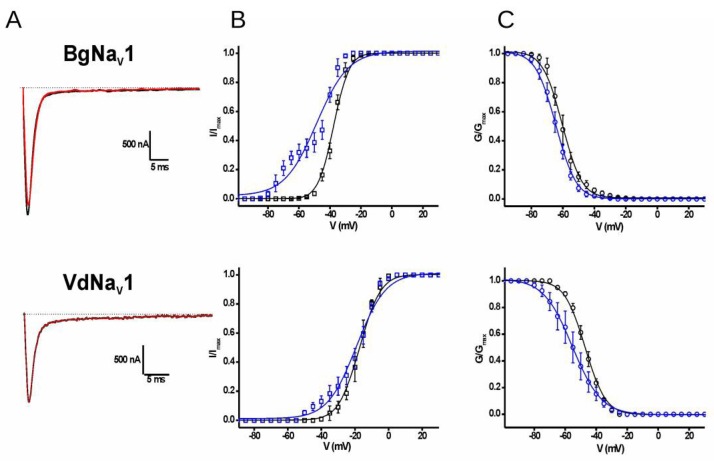
Sodium current traces obtained in BgNa_V_1 and VdNa_V_1 channels. (**A**) Current trace obtained from the experiments. Black traces represent the control condition and red traces in the presence of Tf1a for each subtype. (**B**) Graphs from the activation phase for each subtype. Black squares represent the control condition and blue squares the condition in the presence of the toxin Tf1a. (**C**) Graphs from the inactivation phase. Black circles represent the control condition and blue circles the condition in the presence of Tf1a. The bars represent the standard error of mean. The curves in A and B were done using the Boltzmann modified function.

The toxin Tf1a was also tested on insect (BgNa_V_1 from *Blattella germanica*) and arachnidan (VdNa_V_1 from *Varoa destructor*) sodium channel subtypes at 100 nM final concentration. The toxin was not capable to decrease the peak current at none of the subtypes tested. However, Tf1a affected the conductance-voltage (g-V) during the activation process, with a left shift of the open probability on both channels (ΔV_g_ ≅ −11 mV for BgNa_V_1 and ΔV_g_ ≅ −1.8 mV for VdNa_V_). Tf1a was also capable to affect the steady-state inactivation curves on both subtypes, such as human isoforms (ΔV_h_ ≅ −4.1 mV for BgNa_V_1 and ΔV_h_ ≅ −8.3 mV for VdNa_V_) (Table 4, Figure 7).

## 3. Discussion

In this work a new toxin, Tf1a, was purified and identified from the venom of *Tityus fasciolatus*. Compared to other toxins previously described from different scorpion species, Tf1a revealed a high identity with β-NaScTx, including the peptide called Tf1 also from *Tityus fasciolatus* [5]. The differences between Tf1 and Tf1a can be seen in position 26, an arginine (R) in Tf1 for a serine (S) in Tf1a, and 29, alanine (A) in Tf1 for a lysine (K) in Tf1a, considering the numbering represented in Figure 3. This variability can be a result of many genetics phenomenon such as polymorphism, duplication and trans-splicing, that are pointed to give rise to scorpion toxin diversification [25].

β-NaScTxs hold conservative sequence characteristics, such as the position of cysteine (C), as shown in Figure 3. According to UNIPROT data and peptide similarities, these compounds are linked in a consensus arrangement, C1-C8, C2-C5, C3-C6, and C4-C7, which is an important feature for β-NaScTxs [6,26]. Besides the cysteine position, other regions are highly conserved and important for toxin activity, such as the pharmacophore region (E26 flanked by the hydrophobic residues L13, Y22, and I29), a solvent-exposed aromatic cluster (Y4, Y36, W39, Y43, and Y45), residues located in the N-groove region and some conserved residues in the C-terminal (W54), using Ts1 positions as reference [6,26]. Each one of these regions has an important role in the interaction and activity in sodium channels [6,26]. Most of these regions can be observed in all the sequences shown in Figure 3 including Tf1a, as expected for a β-NaScTxs toxin. 

Some toxins presented in Figure 3 have already been characterized by electrophysiological experiments. Tb1 from *Tityus bahiensis* is the toxin with higher identity (97%); no electrophysiological experiments have been done up to date. Tt1g from *T. trivittatus*, with 96% identity, was tested on Na_V_1.1 to 1.6 stably expressed in HEK 293 cells, and was capable of affecting the open probability on isoforms Na_V_1.2 and 1.3 and reducing the macrocurrents in Na_V_1.4 and 1.5 without changing the voltage dependence at 500 nM [27]. Ts1 from *T. serrulatus* (92% identity) was tested on Na_V_1.1–1.8, DmNa_V_1 and NaChBac expressed in *Xenopus laevis* oocytes, and affected the open probability of Na_V_1.2, 1.3, 1.4, and 1.6, being more effective on Na_V_1.3 and 1.6. The macrocurrents decreased in Na_V_1.3, 1.4, 1.5, and 1.6, with a higher effect in Na_V_1.4 and 1.5 [23]. 

Tf2 (73% identity with Tf1a), the first β-NaScTx described from *Tityus fasciolatus* venom, was tested on Na_V_1.1–1.8 expressed in *Xenopus laevis*. At 1 µM, this toxin was capable to alter the open probability in Na_V_1.3, being ineffective to other isoforms tested [20]. Toxin Tz1 from *Tityus zulianus* (64% identity with Tf1a) was tested on Na_V_1.2, 1.4, 1.5, 1.6, and 1.7 expressed in HEK cells. Tz1 modified more notably the activation kinetics of Na_V_1.4 subtype, whereas Na_V_1.4 and 1.5 macrocurrents were inhibited [28,29,30]. Bactridine 2 from *Tityus discrepans* (64% identity with Tf1a) was tested on Na_V_1.2–1.8, DmNa_V_1, and NaChBac being capable to modulate activation kinetics of Na_V_1.2, 1.4, and 1.6 and inhibited the macrocurrents of Na_V_1.2 and 1.4 at 100 nM, also promoting sodium efflux in gram negative bacteria [31,32]. 

Tpa2 from *Tityus pachyurus* (62% identity with Tf1a) weakly altered Na_V_ channels activity in F11 and TE671 cells at 500 nM [33]. At last, To4 from *T. obscurus* tested on Na_V_1.1–1.7 stably express in HEK 293 cells provoked a weakly macrocurrent inhibition at 70 nM in all isoforms tested. In addition, at 500 nM, To4 shifted the channel open probability to more hyperpolarized voltage and increased the macrocurrent inhibition in Na_V_1.1, 1.2 and 1.4 [24]. 

Comparing Tf1a with the β-NaScTx toxins described above whose the current inhibition has been evaluated on Na_V_, it is possible to observe that most of these toxins (Tt1g, Ts1, Tz1, Bactridine 2, and To4) affect more notably the macrocurrents in Na_V_1.4 and 1.5, except Bactridine 2 that affects only Na_V_1.4, indicating that these subtypes could be more sensitive to macrocurrents inhibition by β toxins interactions. β-toxins are capable to enhance the activation, resulting in the influx of sodium during resting potentials, and the decrease of macrocurrents in strong depolarizations, as shown by Tf1a (Figure S4), Tz1, Ts1, and To4 in the tested Na_V_ isoforms [23,24,29,30]. As described in the voltage-sensor trapping model, the toxin binding enhances closed-state inactivation, stabilizing a partially activated closed state, causing inhibition [14]. When the results from voltage dependence activation are compared, most of these toxins act in specific sodium channels isoforms; differently from Tf1a and To4 that act on all subtypes tested, even with minor effects on each subtype. These differences can be due to sequence variations among these toxins that alter the interaction and activity in specific subtypes of sodium channels as seen in Figure 3. Among Na_V_s isoforms tested in present work, the major effect of Tf1a was the open probability modification of the BgNa_V_1 from the *Blattella germanica* cockroach. The data obtained from human Na_V_ isoforms showed a weak effect on open probability associated with macrocurrent inhibition, while in BgNa_V_1 Tf1a provoked a robust change in kinetic activation without current inhibition. Although not assayed on BgNa_V_1 and VdNa_V_1, Ts1 was active on the insect sodium channel DmNa_V_1 shifting the open probability and changing the current–voltage curves into a bell-shaped relationship [23]. The repertoire of blanks and mode of actions of Tf1a give this toxin two crucial roles to its bearer: defense against mammal predators and incapacitation of insect and Arachnida preys.

In 1998, Cestèle and colleagues proposed a model where the voltage sensor from domain II is trapped by the β-toxin in its outward activated position, preventing its inward movement [14]. This incite the channel to enter in an enhanced-activated condition, facilitating the subsequent depolarizations, making the channel active in a more hyperpolarized potential [34,35]. *Tityus fasciolatus* crude venom induced ECG changes on Winstar rats, including sinus arrhythmia, increased cardiac frequencies, and premature supraventricular complexes [4]. The last two effects indicate hyperactivation states where an enhanced open probability of Na_V_1.5 could play a crucial role. The effect of Tf1a on the activation of Na_V_1.5 could contribute to the enhanced cardiac excitability caused by *T. fasciolatus* venom. 

Mutations on genes responsible for the expression of Na_V_1.5 (SCN5A) can cause heart diseases such as Long QT syndrome type III, Brugada and other cardiopathies [17,18]. These diseases tend to cause a gain or a loss of function, altering the normal functioning and kinetics of the sodium channels present in the regions of the heart [36]. Among 400 mutations described for Na_V_1.5 that cause any kind of disease, about 50% are responsible for causing Brugada syndrome and 30% for Long QT syndrome type III [18]. These mutations tend to happen in transmembrane regions, which can cause some disturbance on voltage sensing or sodium conductance [18]. Considering the effects of *Tityus fasciolatus* crude venom in heart activity in rats and the effects of Tf1a in the kinetics of hNa_V_1.5, could be promising to evaluate the effects of this toxin in mutated variants of Na_V_1.5 related to loss-of-function heart diseases. Although the promiscuous activity of Tf1a among sodium channels isoforms, this toxin is the first β-toxin capable to displace a shift on the open probability in Na_V_1.5. The importance of Tf1as as pharmacological tool needs to be more explored. 

It was previously described that a previous stimulation or prepulse was fundamental to expose the voltage sensor (S4) to allow the interaction with the β-toxin CssIV [14]. However, Campos and colleagues showed that the prepulse was not necessary for β-toxin Ts1 activity [37]. Campos and colleagues also demonstrated by using fluorescence that Ts1 could maintain the voltage sensor in the active position, contributing to elucidate the activity of these toxins [37]. These previous studies (with Ts1 and CssIV) present strong experimental support for the use of prepulse, reinforcing the prudent strategy to keep testing the role of the prepulse when evaluating β-toxin activity. The effects induced by Tf1a in Na_V_ isoforms tested were not changed by the absence or presence of a prepulse.

## 4. Conclusions

In this work a new β-toxin purified from the venom of *Tityus fasciolatus* was electrophysiologically characterized on diverse voltage gated sodium channel subtypes from human, insect, and Arachnida. It was shown that the toxin Tf1a can modify the gating behavior and kinetics of the channels tested, contributing to the understanding of the activity of β-NaScTxs on voltage gated sodium channels.

## 5. Materials and Methods 

### 5.1. Animals Capture and Venom Extraction 

*Tityus fasciolatus* specimens were captured in Brasilia, Federal District, Brazil, under license No. 19138-1 (IBAMA—Instituto Brasileiro do Meio Ambiente e dos Recursos Naturais). The animals were maintained in a proper facility at the University of Brasilia with food and water ad libitum. Crude venom was extracted by electric stimulation of the telson, collected in trifluoroacetic acid (TFA) 0.12% solution and centrifuged at 15,000× *g* for 15 min. The supernatant was collected, quantified at 280 nm and dried as described in [20].

### 5.2. Toxin Purification

Crude venom of *Tityus fasciolatus* was fractioned by RP-HPLC (Reversed Phase High Performance Liquid Chromatography) (Shimadzu Co., Kyoto, Japan), using a C18 column (Synergi Fusion RP 4 μ, 80 Å, 250 × 4.6 mm (Phenomenex, Inc., Torrance, CA, USA). Components were separated using a linear gradient of solvent A (0.12% TFA in water) and solvent B (0.10% TFA in acetonitrile) from 0 to 60% for 60 min at a 1 mL/min flow rate as described previously [20]. Three extra steps of RP-HPLC were conducted to purify Tf1a, the first with 0.5%[B]/min, second purification step with 0.5%[B]/min at 45 °C, and the last purification step with 0.3%[B]/min at 45 °C.

### 5.3. Molecular Mass and Partial Sequence Determination

Molecular mass analyses were made with AutoFlex Speed MALDI TOF/TOF (Bruker Daltonics, Ettlingen, Germany). The sample was diluted in an α-cyano- 4-hydroxycinnamic acid matrix (1:3; *v:v*) plated and analyzed in linear mode. The partial amino acid sequence was obtained by In Source Decay (ISD) method using 1,5-diaminonaphthalene (DAN) solution (1:1; *v:v*). Sequencing and data analysis were conducted with FlexAnalysis 3.4 (Bruker Daltonics, Ettlingen, Germany). Molecular mass and sequence obtained were compared to data acquired from previously constructed RNA library from *Tityus fasciolatus* venom gland (not published data). Similarity was obtained BLAST search (www.ncbi.nlm.nih.gov/blast) and the sequences with higher identities with Tf1a were aligned with Clustal Omega (http://www.ebi.ac.uk/Tools/msa/clustalo/). 

### 5.4. Electrophysiological Assays

#### 5.4.1. Human Channels (hNa_V_)

##### Cell Culture

The cells expressing several Na_V_s isoforms were a kind gift from Dra. Rita Restano-Cassulini from Biotechnology Institute-UNAM (Mexico). Human Embryonic Kidney 293 (HEK) cells expressing hNa_V_1.1–1.6 and Chinese Hamster Ovary (CHO) expressing hNa_V_1.7 were cultivated in DMEN medium (GIBCO, Waltham, MA, USA) complemented with 4.5% Glucose, 10% Fetal Bovine Serum, and G418 antibiotic (0.5 mg/mL). For HEK cells, 1% MEM Non-Essential Amino Acid Solution were also added into the medium and G418 antibiotic (0.4 mg/mL). The cells were grown at 37 °C with 5% CO_2_ and carried every 48 h. The cells were cultured using all safety procedures to avoid any contamination. Auxiliary β1A subunit are endogenous expressed in HEK cells and there are experimental evidences of coassembling of heterologous Na_V_ channels and endogenous β1A subunits in HEK [38].

##### Human Na_V_ Subtypes Current Recording

The experiments were performed using whole cell patch-clamp technique in a HEKA EPC 10 amplifier and Patchmaster software (HEKA Elektronik, Lambrecht/Pfalz, Germany). The pipettes made of borosilicate glass forged in a horizontal puller P97 (Sutter Instruments, Novato, CA, USA) had resistance between 1.5–3 MΩ after filled with internal solution. Internal solution was composed by (mM): CsF 105, CsCl 27, NaCl 5, MgCl_2_ 2, EGTA 10, HEPES 10, pH 7.3 corrected with CsOH. The external solution used for the experiments was made of (mM): NaCl 130, KCl 5, CaCl_2_.2H_2_O 2, MgCl_2_·6H_2_O 2, HEPES 10, and glucose 10, pH 7.4 adjusted with NaOH. The series resistance during all the experiments was ~10 MΩ that was compensated at 70%. The p/−4 protocol with a hold potential of −120 was applied to cancel the capacitive and leak currents.

The electrophysiological assays were performed using a three-step protocol where the cells are maintained at a holding potential of −100 mV and submitted to a prepulse of 30 mV for 5 ms, then back to the holding potential again by 30 ms. After that, voltage steps varying from −90 to 15 mV were performed with increment of 5 mV at each sweep with an interval of 2 s. Immediately after the end of each sweep, a stimulus of −10 mV was applied to evaluate the steady-state inactivation process. Initially, cells were submitted to this procedure without the presence of the toxin for approximately five minutes being this procedure the control experiment. After obtaining stable control recordings for each cell, a final concentration of 100 nM of toxin was added and the effects were recorded for 10 min. The experiments were all made at room temperature (~24 °C). 

For the recovery from inactivation, currents were obtained at two-pulse protocol, where a 10 ms prepulse to −10 mV was done, followed by resting at −100 mV with an incremental time interval between the two pulses of 2 ms by cycle, varying from 2 to 80 ms and a test pulse to −10 mV for 20 ms. 

##### Data Analysis

The parameters evaluated to determine Tf1a effects on sodium channels were the uninhibited fraction of current (Fu), recovery from inactivation and the voltage shift in activation and steady-state inactivation (SSI). The sodium conductance (gNa) was calculated from the currents according to Ohm’s law:

(1) $$\;\mathrm{gNa}\;=\;\frac{\mathrm{INa}\;}{\left(V-\mathrm{Vrev}\right)}$$

where V represents the test potential that triggers the peak current INa the Na^+^ current peak amplitude at a given V, and Vrev is the reversal potential calculated from Nernst equation:

(2) $$\;\mathrm{Vrev}=\;\frac{\mathrm{RT}\;}{\mathrm{zF}}\ln\frac{\left[\mathrm{Na}\right]e}{\left[\mathrm{Na}\right]i}$$

where R represent the gas constant, T is the temperature in absolute temperature in Kelvin scale, z is the ion valence, F is the Faraday constant, and [Na]e, [Na]i correspond to the concentration of sodium in external and internal solution used in the experiments.

The data converted in gNa from activation process were normalized to the maximal Na^+^ conductance amplitude and fitted in a single Boltzmann’s function to evaluate the fraction open channels ( ρO):

(3) $$\;\rho O=\;\frac{1}{1+\exp\left[\left(\frac{V-V_{g}\;}{k}\right)\right]}$$

where V_g_ is the voltage in which half of the Na_V_ channels are in the open state and k is the slope factor.

The ionic current data obtained from stimulus protocol designated for steady-state inactivation were normalized to the maximal Na^+^ current amplitude and plotted against prepulse potential and fitted in a single Boltzmann’s function:

(4) $$\;\mathrm{SSI}=\;\frac{1}{1+\exp\left[\left(\frac{V-V_{h}\;}{k}\right)\right]}$$

where V_h_ is the voltage in which half of of Na_V_ channels remaining open.

The current fraction recovering obtained from the recovery protocol was plotted against the interval time and fitted in a single exponential function:

(5) $$\;y=y0+y1\left(1-e^{-\frac{t}{\tau }\;}\right)$$

where y0 is the amplitude in each time t, y1 is the final amplitude, t is the time, and τ is the time constant. The time constant (τ) in the absence and presence of toxin as compared. 

Statistical analyses were performed with V_g_, V_h_, and τ data using Graph Pad Prism 5.01 (GraphPad software, La Jotta, CA, USA, 2007). Standard two-tailed paired Student’s *t*-test were used to compare the different values and considered significant at *p* < 0.05. 

#### 5.4.2. Insect and Arachnida Channel Subtypes—Expression of Voltage-Gated Ion Channels in Xenopus Laevis Oocytes

For the expression of the insect channel BgNa_V_1, the arachnid channel VdNa_V_1, and the auxiliary subunit TipE in *Xenopus* oocytes, the linearized plasmids were transcribed using the T7 or SP6 mMessage-mMachine transcription kit (Ambion, Carlsbad, CA, USA). The harvesting of stage V–VI oocytes from anesthetized female *Xenopus laevis* frogs was previously described [39]. Oocytes were injected with 50 nL of cRNA at a concentration of 1 ng/nL using a microinjector (Drummond Scientific, Broomall, PA, USA). The oocytes were incubated in a solution containing (in mM) 96 NaCl, 2 KCl, 1.8 CaCl_2_, 2 MgCl_2_, and 5 HEPES (pH 7.4), supplemented with 50 μg/mL gentamicin sulfate [23].

##### Insect and Arachnida Channels Recordings

Experiments were performed using two-electrode voltage-clamp recordings at room temperature (18–22 °C) using a Geneclamp 500 amplifier (Molecular Devices, Downingtown, PA, USA) controlled by a pClamp data acquisition system (Axon Instruments, Union City, CA, USA). Whole-cell currents from oocytes were recorded 1−4 days after injection. Bath solution composition was the following (in mM): 96 NaCl, 2 KCl, 1.8 CaCl_2_, 2 MgCl_2_, and 5 HEPES (pH 7.4). Voltage and current electrodes were filled with 3 M KCl.

Resistances of both electrodes were kept between 0.8 and 1.5 MΩ. The elicited currents were filtered at 2 kHz and sampled at 20 kHz using a 4-pole low-pass Bessel filter. Leak subtraction was performed using a −P/4 protocol. To avoid overestimation of a potential toxin-induced shift in the current–voltage relationships of inadequate voltage control when measuring large sodium currents in oocytes; only data obtained from cells exhibiting currents with peak amplitude <2 μA were considered for analysis. For the electrophysiological analysis, a number of protocols were applied from a holding potential of −90 mV with a start-to-start interval of 0.2 Hz. Sodium current traces were evoked by 100-ms depolarizations to Vmax (the voltage corresponding to maximal sodium current in control conditions). The current–voltage relationships were determined by 50-ms step depolarizations between −90 and 70 mV, using 5-mV increments as previously described [23,40].

##### Insect and Arachnida Data Analysis

The sodium conductance (gNa) and 𝜌𝑂 curves were calculated in a similar way by the formulas 1 and 3. Toxin-induced effects on the steady-state inactivation were investigated using a standard 2-step protocol. In this protocol, 100-ms conditioning 5-mV step prepulses ranging from −90 to 70 mV were followed by a 50-ms test pulse to −30 or −10 mV.

Data were normalized to the maximal Na^+^ current amplitude, plotted against prepulse potential, and fitted using a single Boltzmann equation:

(6) $$\;\frac{I_{\mathrm{Na}\;}}{I_{\max}}=\left[\frac{1-C}{1+\exp\left(\frac{V-V_{h})}{k_{h}}\right)}\right]+C$$

where Imax is the maximal INa, V_h_ is the voltage corresponding to half-maximal inactivation, V is the test voltage, k is the slope factor, and C is a constant representing a non-inactivating persistent fraction (close to 0 in control).

All data are presented as means ± SE of ≥6 independent experiments (*n* ≥ 6). All data were analyzed using pClamp Clampfit 10.4 (Molecular Devices, San Jose, CA, USA, 2013) and Origin 7.5 software (Originlab Corp., Northampton, MA, USA, 2003).

## Figures and Tables

**Table 1:** Open probability (ρO) of the activation phase and uninhibited fraction current (Fu). V_g_ is the voltage corresponding to half-maximal activation; k is the voltage steepness (slope) in activation. Data are represented by mean and standard error.

	V_g_ Control (mV)	V_g_ Toxin (mV)	k Control (mV)	k Toxin (mV)	Fu
	Mean	Mean	Mean	Mean	Mean
**hNa_V_1.1**	−20.21 ± 2.24	−27.30 ± 2.51	4.29 ± 0.54	4.53 ± 0.41	0.83 ± 0.03
**hNa_V_1.2**	−17.21 ± 1.71	−23.30 ± 1.61	4.89 ± 0.35	5.55 ± 0.08	0.70 ± 0.02
**hNa_V_1.3**	−12.91 ± 1.98	−17.70±2.12	6.93 ± 0.46	7.38 ± 0.52	0.83 ± 0.06
**hNa_V_1.4**	−18.41 ± 1.69	−26.34 ± 2.93	6.71 ± 0.35	6.61 ± 0.46	0.47 ± 0.07
**hNa_V_1.5**	−36.62 ± 2.10	−42.91 ± 1.72	5.95 ± 0.37	6.40 ± 0.41	0.29 ± 0.03
**hNa_V_1.6**	−23.40 ± 2.01	−32.41 ± 2.80	5.70 ± 0.58	5.72 ± 0.29	0.77 ± 0.10
**hNa_V_1.7**	−18 ± 2.69	−25.93 ± 1.84	5.90 ± 0.66	5.83 ± 0.53	0.80 ± 0.14

**Table 2:** Parameters for steady state inactivation (SSI). V_h_ is the voltage corresponding to half-maximal inactivation; k_h_ is the voltage steepness (slope) in inactivation. Data are represented by mean and standard error.

	V_h_ Control (mV)	V_h_ Toxin (mV)	k_h_ Control (mV)	k_h_ Toxin (mV)
	Mean	Mean	Mean	Mean
**hNa_V_1.1**	−45.92 ± 1.17	−54.03 ± 2.24	8.48 ± 0.28	8.6 ± 0.12
**hNa_V_1.2**	−46.92 ± 1.22	−52.29 ± 0.94	7.98 ± 0.32	8.45 ± 0.49
**hNa_V_1.3**	−47.57 ± 1.52	−50.5 ± 1.71	11.34 ± 0.57	9.92 ± 1.02
**hNa_V_1.4**	−68.15 ± 1.22	−74.31 ± 2.25	8.19 ± 0.43	8.25 ± 0.31
**hNa_V_1.5**	−66.18 ± 0.99	−72.71 ± 1.43	9.38 ± 0.47	7.85 ± 0.41
**hNa_V_1.6**	−55.78 ± 1.90	−67.19 ± 2.52	8.03 ± 0.26	8.61 ± 0.64
**hNa_V_1.7**	−47.46 ± 3.85	−55.45 ± 6.04	11.20 ± 1.32	11.20 ± 1.70

**Table 3:** Recovery from inactivation obtained for the Na_V_ subtypes tested. Data are represented by mean and standard error. *n* represent the number of independent measures.

		τ Control (ms)	τ Toxin (ms)
	*n*	Mean	Mean
**hNa_V_1.1**	3	3.01 ± 0.27	4.07 ± 0.41
**hNa_V_1.2**	4	2.42 ± 0.16	3.90 ± 0.30
**hNa_V_1.3**	4	10.12 ± 2.62	12.55 ± 3.97
**hNa_V_1.4**	4	4.33 ± 0.37	5.21 ± 0.48
**hNa_V_1.5**	3	25.0 ± 5.62	45.11 ± 3.99
**hNa_V_1.6**	4	11.53 ± 1.30	12.76 ± 2.22
**hNa_V_1.7**	4	24.35 ± 0.89	29.65 ± 2.20

**Table 4:** Parameters from activation phase and steady-state inactivation on insect (BgNa_V_1) and arachnidan (VdNa_V_1) channels. V_g_ is the voltage corresponding to half-maximal activation; V_h_ is the voltage corresponding to half-maximal inactivation. Data are represented with mean and standard error. *n* = 6.

	V_g_ Control (mV)	V_g_ Toxin (mV)	V_h_ Toxin (mV)	V_h_ Toxin (mV)
	Mean	Mean	Mean	Mean
**BgNa_V_1**	−37.6 ± 0.1	−48.6 ± 1.2	−60.3 ± 0.2	−64.4 ± 0.1
**VdNa_V_1**	−17.2 ± 0.1	−19.0 ± 0.5	−47.2 ± 0.1	−55.5 ± 0.2

## Supplementary Materials

The following are available online at http://www.mdpi.com/2072-6651/10/9/339/s1, Figure S1: Average mass ([M + H]^+^) of the fraction corresponding to the toxin Tf1a; Figure S2: Partial sequence from the fraction of interest corresponding to the toxin Tf1a; Figure S3: Comparison between RNA library sequence and the partial sequence obtained by ISD method. Figure S4: Voltage-current relationship (IV) from human Na_V_ isoforms tested. Red traces represent the presence of 100 nM of Tf1a and black trace the control condition; Table S1: Open probability (ρO) of the activation phase without prepulse. V_g_ is the voltage corresponding to half-maximal activation in the experiments without use of prepulse.
