# Supplementary material for: Subtype Specificity of β-Toxin Tf1a from Tityus fasciolatus in Voltage Gated Sodium Channels
Source: Toxins (Basel). 2018 Aug 22;10(9):339. doi: 10.3390/toxins10090339 (PMC6162530; doi:10.3390/toxins10090339)
Supplement: Supplementary file 1 [file toxins-10-00339-s001.pdf]

Supplementary Materials: Subtype Specificity of β-Toxin Tf1a from *Tityus fasciolatus* in Voltage Gated Sodium Channels

Daniel Oliveira da Mata, Diogo Vieira Tibery, Leandro Ambrósio Campos,

Thalita Soares Camargos, Steve Peigneur, Jan Tytgat and Elisabeth Ferroni Schwartz

Table S1. Open probability (ϱO) of the activation phase without prepulse. V_g is the voltage corresponding to half−maximal activation in the experiments without use of prepulse. Data are represented by mean and standard error.

	V_g Control (mV) Mean	V_g Toxin (mV) Mean
**HNav1.1**	−20.23 ± 2.27	−27.92 ± 2.99
**hNav1.2**	−17.21 ± 1.90	−23.97 ± 1.53
**hNav1.3**	−12.94 ± 2.14	−16.60± 1.80
**hNav1.4**	−18.86 ± 1.64	−25.83 ± 3.42
**hNav1.5**	−39.57 ± 2.53	−43.30 ± 2.38
**hNav1.6**	−24.16 ± 2.63	−32.52 ± 2.77
**hNav1.7**	−14.50 ± 4.21	−23.28 ± 2.76

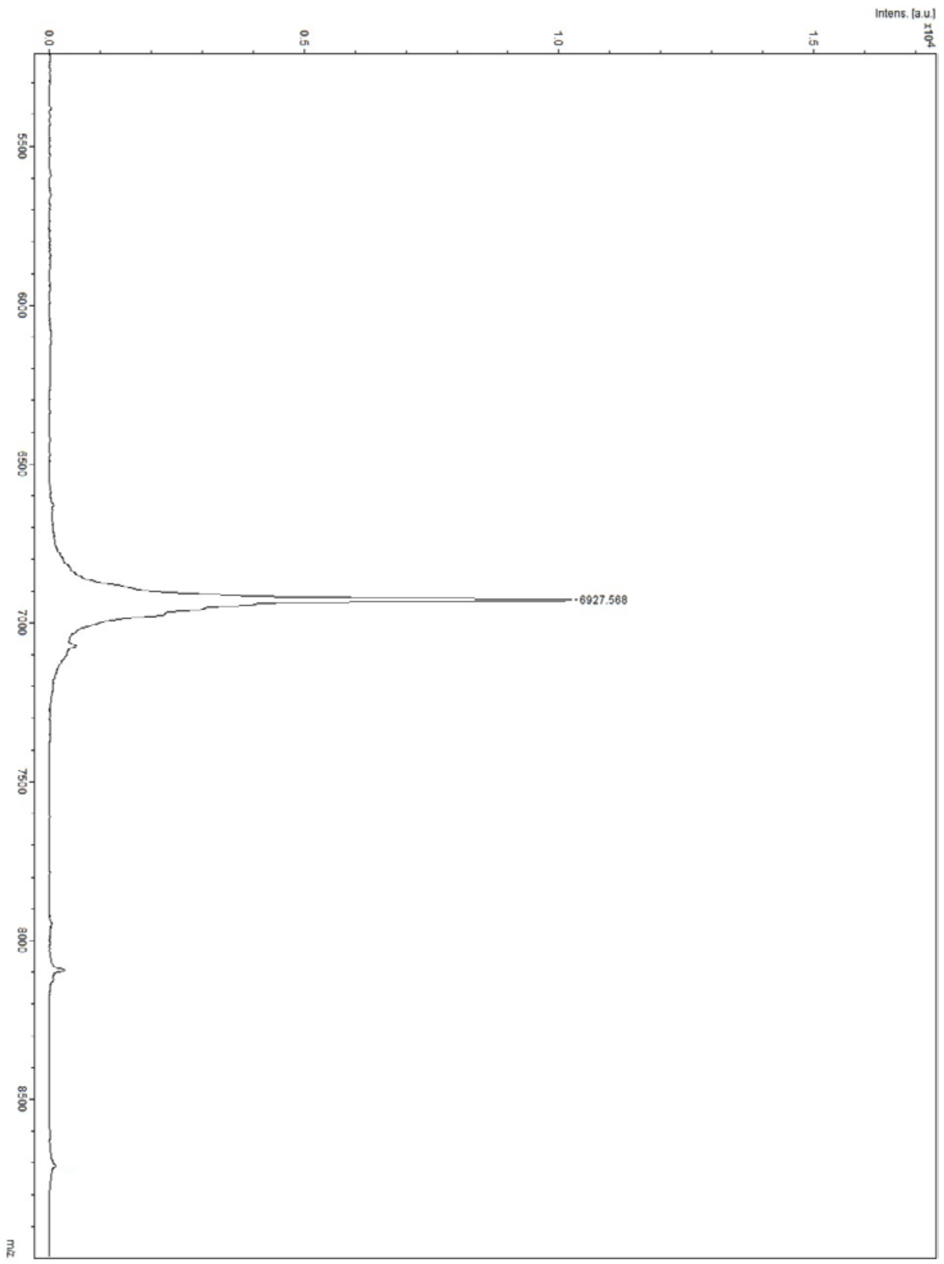

Figure S1. Average mass ($[M+H]^+$) of the fraction corresponding to the toxin Tf1a.

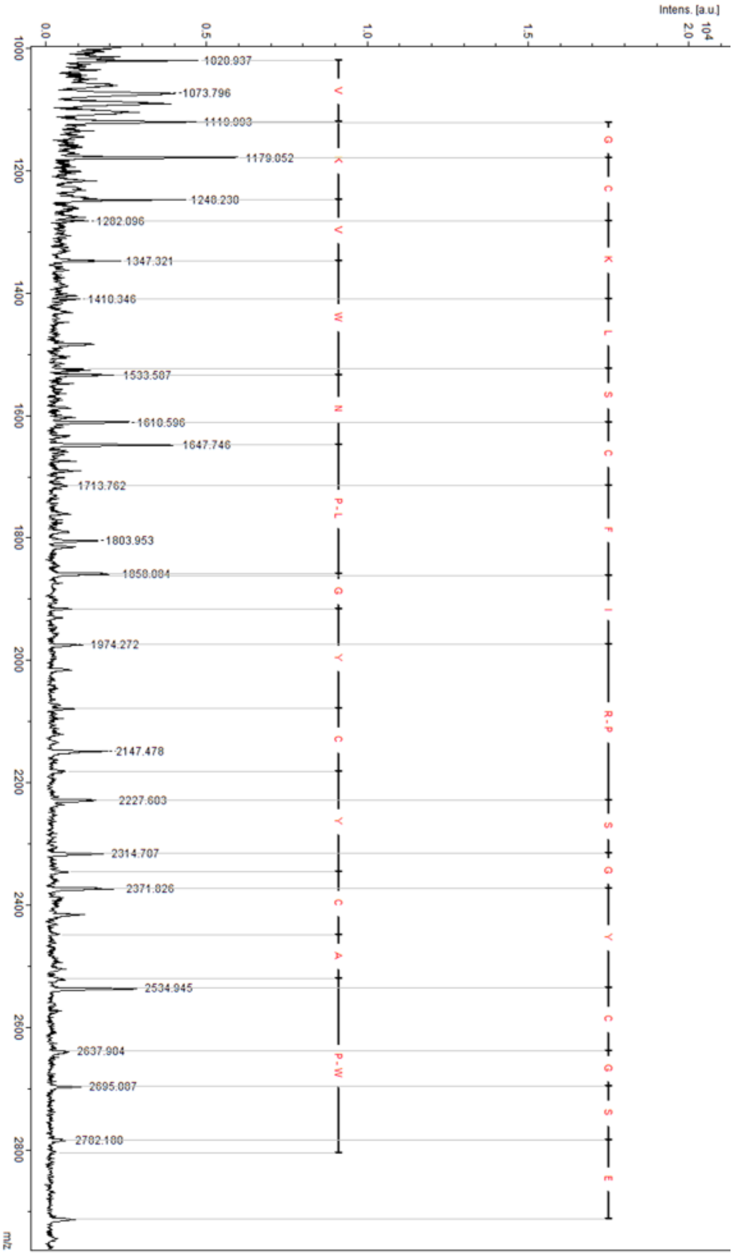

Figure S2. Partial sequence of the fraction of interest corresponding to the toxin Tf1a.

```
RNA Libary Sequence- KEGYLMDHEGCKLSCFIRPSGYCGSECKIKKGSSGYCAWPACYCYGLPNWVKVWERATNRC
   Partial Sequence- ---------GCKLSCFIRPSGYCGSE------------WPACYCYGLPNWVKV--------
```

Figure S3. Comparison between RNA library sequence and the partial sequence obtained by ISD method.

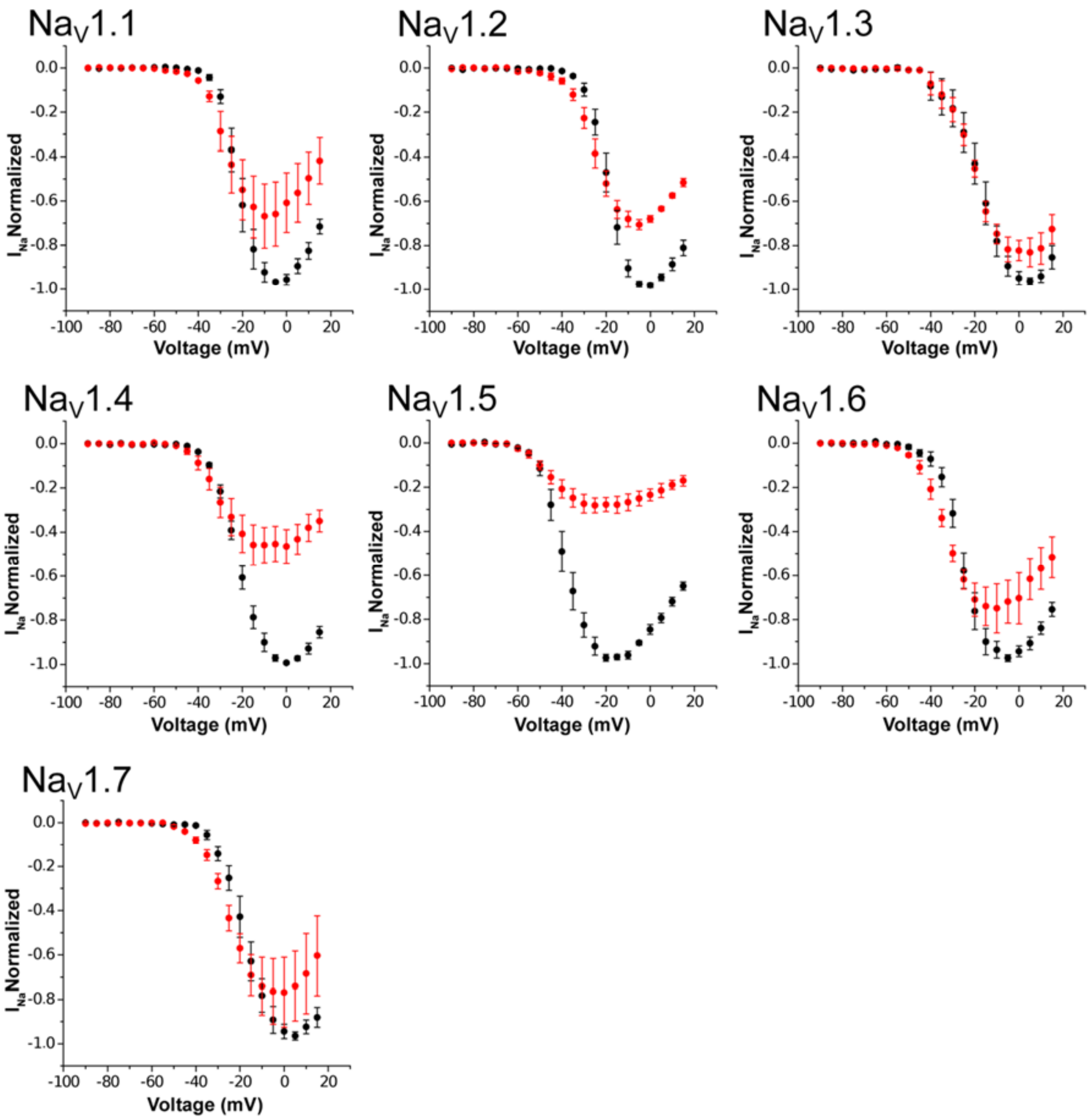

Figure S4. Voltage-current relationship (IV) from human Na$_V$ isoforms tested. Red traces represent the presence of 100 nM of Tf1a and black trace the control condition.
